# Development and initial validation of the multilingual Swiss version of the brief parental burnout scale

**DOI:** 10.1177/20551029241308777

**Published:** 2024-12-20

**Authors:** Seraina Caviezel Schmitz, Paula Krüger

**Affiliations:** 30744 Lucerne University of Applied Sciences and Arts, Switzerland

**Keywords:** Family, burnout, depression, validation, scale, prevalence

## Abstract

Parental burnout (PB) has adverse effects on parents and children. The aim of the present study was to develop a multilingual version of the Brief Parental Burnout Scale (BPBS) adapted to the Swiss context in order to (1) provide practitioners working with families in different language regions of Switzerland with a screening tool, and (2) to estimate initial prevalence rates for risk of PB. Overall, results indicate, that the Trilingual Swiss BPBS (3-L-BPBS) is a promising screening tool, allowing for early detection of parents at risk and therefore for the prevention of negative consequences of PB. Further, results suggests that a substantial proportion of Swiss parents is at moderate to high risk for PB, with prevalence rates varying between language regions. However, additional research is needed to further validate the 3-L-BPBS and to deepen our understanding of the impact of structural and cultural differences on PB.

## Introduction

Raising and caring for children is associated with challenges and stress. However, parental burnout exceeds typical transient parenting stress (Mikolajczak et al., 2021). We speak of parental burnout when the parents’ stress in their parental role clearly and chronically exceeds their resources. Parental burnout is characterised by three aspects: (i.) an intense emotional exhaustion regarding the parental role, (ii.) emotional distancing from one’s own children, and (iii.) loss of fulfilment in the parental role and of energy to carry it out, which stands in contrast to the parents’ previous emotional state (Mikolajczak et al., 2019).

Although parental burnout is still much less well researched than job burnout, research on the concept itself, prevalence rates and outcomes of parental burnout is growing (e.g. Mikolajczak et al., 2018a, 2018b, 2020; Roskam et al., 2021; Sorkkila and Aunola, 2020). For example, studies have shown an association between depression and both parental and job burnout. Further, depression and burnout are associated with somatic complaints, sleep disorders, alcohol abuse, couple conflicts, and suicidal ideation (Cai et al., 2021; Edú-Valsania et al., 2022; Mikolajczak et al., 2018a, 2019; Oh et al., 2023). But despite these commonalities, Mikolajczak et al. (2020) were able to show that even though parental burnout, job burnout and depression are intersecting concepts, they are not identical. For example, job and parental burnout each have specific outcomes associated with them which cannot be explained by depressive symptoms, such as the intention to leave the company or negative parenting behaviours, including child abuse and neglect (Aunola et al., 2021; Hansotte et al., 2021; Mikolajczak et al., 2018a, 2019, 2020). However, regarding possible outcomes of parental burnout it must be noted, that the majority of studies conducted so far are cross-sectional without control groups. Therefore, no causal conclusions can be drawn.

### Prevalence of parental burnout

In recent years, prevalence rates for parental burnout have been assessed in different countries. Roskam et al. (2021) report prevalence rates for parental burnout from 42 countries, noting, among other things, that higher rates were found in Western, individualistic cultures in comparison to rates in more collectivistic cultures. But even prevalence rates for European countries vary significantly. Based on non-representative samples, varying prevalence rates for parental burnout were reported for Switzerland’s neighbouring countries France (5.3–6.2%; *n* = 1357), Austria (1.6–1.8%; *n* = 185), Germany (1.5–1.8%; *n* = 204), and Italy (0.5–1.1%; *n* = 350). Rates depending on the cut-off score used or sample corrections (see Roskam et al., 2021). For Switzerland, reported prevalence rates for parental burnout vary between 3.2% and 7.1%. However, these rates were not only based on a non-representative sample of 419 parents, participants were also exclusively from the canton of Vaud (Roskam et al., 2021; see also Favez et al., 2023), which is one of 26 Swiss cantons located in the French-speaking part of Switzerland (“Romandy”), representing 9% of the Swiss population (Federal Statistical Office, 2023a). Hence, these figures cannot be readily generalised to Switzerland as a whole for three reasons in particular: First, data are based on a sample that is neither representative for the canton of Vaud nor for all 26 cantons. Second, there are structural and cultural differences between the language regions of Switzerland regarding family-related issues. For example, non-traditional household forms, such as lone parents with child(ren), are more common in French-speaking Switzerland than in the other two language regions. In addition, women in German-speaking Switzerland are more often responsible for domestic work and childcare, and consequently less often working full-time than their male partners (Federal Statistical Office, 2021). Correspondingly, extrafamilial supplementary childcare is used significantly more often in Romandy than in German- or Italian-speaking Switzerland, where instead grandparents are much more involved in childcare (Federal Statistical Office, 2020, 2021). In accordance with that, approval of traditional gender roles is lowest in French-speaking and highest in German-speaking regions (Federal Statistical Office, 2021). Furthermore, regional differences regarding parents’ educational attitudes can be found. For example, corporal punishment of children is less frequently practised on a regular basis by parents in German-speaking Switzerland than in the other language regions (e.g. slapping on the bum with hand: 1.6% vs 3.9% [Romandy] or 5.5% [Italian-speaking canton of Ticino]; Schöbi et al., 2020). Roskam et al. (2021) also stressed the importance of considering cultural differences when researching parental burnout, even though they focused on differences on a national level.

Third, the reported prevalence rates for parental burnout for the canton of Vaud cannot be generalised to today’s Vaud or Switzerland because these data were collected in 2018, before the COVID-19 pandemic (Aunola et al., 2021; Roskam et al., 2021). The COVID-19 pandemic has led to a wide range of challenges and strain for the Swiss population in general and for individual population groups in particular, such as families (e.g. Diaz Hernandez et al., 2021; Fuchs et al., 2021; Refle et al., 2020), the long-term effects of which are not yet fully known. Van Bakel et al. (2022) compared pre- and peri-pandemic prevalence rates for parental burnout across 26 countries, showing a significant increase in parental burnout rates during the pandemic. It is reasonable to assume, that pandemic-related challenges and strain has also led to an increase in parental burnout in Switzerland. However, (language) regions were not equally affected by the pandemic and pandemic-related public health measures. Ticino was hit the hardest, followed by French-speaking Switzerland (e.g. Refle et al., 2020). Therefore, the fall-out of the pandemic is another reason to assume that post-pandemic prevalence rates for parental burnout are not only higher than pre-pandemic rates but also vary across language regions in Switzerland.

### Measuring parental burnout

To identify parents facing parental burnout, two measures are widely used: the Parental Burnout Inventory (PBI; Roskam et al., 2017; 2018b) and the Parental Burnout Assessment (PBA; Roskam et al., 2018a). Both scales are similar, but the authors recommend to use the PBA for research purposes, except when burnout is compared across contexts, that is work and parenting. The PBA consists of 23 items representing four factors: emotional exhaustion in the parental role, feeling fed up with parenting, feeling emotionally distanced from the child(ren), and contrast with former parental self (Roskam et al., 2022). Items are rated on a 7-point scale ranging from 0 (“never”) to 6 (“daily”). The PBA has been shown to be a reliable and valid assessment tool in various cultural contexts (e.g. Aunola et al., 2020; Furutani et al., 2020; Hamvai et al., 2022; Matias et al., 2020; Szczygieł et al., 2020). However, although the PBA can be completed fairly quickly, it is too long to be used for general screening. Therefore, Aunola et al. (2021) developed the Brief Parental Burnout scale (BPBs) consisting of just five items, representing the first three of the above-mentioned factors (Aunola et al., 2021). Initial research suggests that the BPBs is suitable for identifying both parents already facing parental burnout and those at risk (Aunola et al., 2021).

## The current research

Considering the potential adverse outcomes of parental burnout for parents, children and families as well as the fact that up to 7% of Swiss parents might be affected, early detection of parents at risk seems crucial to prevent negative health outcomes for parents and children alike. Therefore, the aim of the study was to develop a multilingual Swiss version of the Brief Parental Burnout Scale and to conduct an initial validation of the scale. Further, the study aimed to estimate initial prevalence rates for parental burnout risk in different language regions of Switzerland. For this purpose, we translated the English version of Aunola et al.’s (2021) Brief Parental Burnout Scale (BPBs) into German, French and Italian and adapted it to the Swiss context.

Building on previous studies (e.g. Aunola et al., 2020; Prandstetter et al., 2023; Szczygieł et al., 2020), we hypothesised that higher parental burnout risk scores would be associated with (i.) lower life satisfaction, (ii.) lower satisfaction with family life, (iii.) higher perceived burden of caring for children or other family members, (iv.) more depressive symptoms (in particular, more sleeping disorders), and (v.) lower social support.

Regarding initial prevalence rates for risk for parental burnout, we expected differences across language regions due to the mentioned structural and cultural differences. However, because almost nothing is known about parental burnout in Switzerland, we did not formulate directed hypotheses.

## Method

### Overview

The analyses presented here are part of the authors’ longitudinal study “Family Violence and COVID-19: The Pandemic Within the Pandemic”. Within the framework of this study, we repeatedly survey an access panel of the polling institute GfS.Bern (Krüger and Caviezel Schmitz, 2022). Results reported here relate to the online survey conducted in Spring 2022, in which the risk for parental burnout was examined for the first time.

### Procedure

As a screening instrument for parental burnout risk in Switzerland, we developed the Trilingual Swiss Brief Parental Burnout Scale (3-L-BPBS). As mentioned above, we translated the English version of Aunola et al.’s (2021) Brief Parental Burnout Scale (BPBs) into German, French and Italian and adapted it to the Swiss context. First, items were translated by the authors of the study. Then, the translation was proofread by two German native speakers, two French native-like speakers and one Italian native speaker. Lastly, the French translation was compared to the BPBs’s French version presented by Aunola et al. (2021). In case of discrepancies between language versions, the one linguistically closer to the BPBs English version was chosen, in order to keep the different language versions as similar as possible.

In May 2022, one month after the Swiss government dropped all pandemic-related public health measures, we conducted an online survey regarding family conflict and violence in Switzerland with the help of the polling institute Gfs.Bern. The questionnaire included questions regarding family violence victimization and perpetration as well as known risk and protective factors for family violence (for example, social support, financial problems) (Krüger and Caviezel Schmitz, 2022). Since parental burnout risk is a potential risk factor for child abuse and neglect as one form of family violence, we added the 3-L-BPBS to the questionnaire in 2022. The 3-L-BPBS was presented to participants who stated that they had at least one (foster/step) child. Informed consent was given by all participants. Participants were assured that their responses remained anonymous. The polling institute did not provide researchers with any information allowing them to identify individual participants.

### Sample

In total, 1766 respondents participated in the survey. 685 participants stated that they had at least one (foster/step) child. 61.2% of these participants answered all five items of the 3-L-BPBS (*n* = 419), but only 237 of these respondents also had minor children (56.6%). Therefore, only 237 parents of minor children were included in the analyses. 59.1% of these parents completed the scale’s German version (*n* = 140), 34.6% the French version (*n* = 82), and 15 respondents completed the Italian version (6.3%). It was therefore to be expected that the majority of respondents lived in German-speaking Switzerland (61.2%), 34.6% in the French-speaking region and the minority of parents in the Italian-speaking canton of Ticino (4.2%). Accordingly, “language version” and “language region” were strongly correlated (Cramer’s V = 0.841, *p* < .001).

The gender ratio was roughly balanced with 49.4% mothers. The participants’ age ranged between 22 and 71, on average they were 42 years old (*M* = 42.57, *Mdn* = 42.00, *SD* = 8.48 years). The vast majority of participants were in a committed relationship (92.0%, *n* = 218), 10.1% were single parents (*n* = 24). Respondents had between 1 and 6 children, with the majority having one child (37.1%, *n* = 88) or two children (46.0%, *n* = 109). On average, their youngest child was 6–7 years old (*M* = 7.30, *Mdn* = 6.00, *SD* = 5.29 years). Further key sociodemographic characteristics of the subsample of parents are described in Table 1.

**Table 1.** Sample characteristics, in total and differentiated by language versions of the 3-L-BPBS (%, n).

	Sample overall (*N* = 237)	German version (*n* = 140)	French version (*n* = 82)	Italian version (*n* = 15)
Educational status				
Compulsory education	3.8% (9)	2.1% (3)	4.9% (4)	13.3% (2)
Upper secondary level education	29.5% (70)	27.9% (39)	29.3% (24)	46.7% (7)
Tertiary level education	66.7% (158)	70.0% (98)	65.9% (54)	40.0% (6)
Household income				
Getting by (comfortably) on household income	84.8% (201)	90.0% (126)	80.5% (66)	60.0% (9)
(Very) difficult to get by on household income	15.2% (36)	10.0% (14)	19.5% (16)	40.0% (6)
Relationship status				
Committed relationship	92.0% (218)	95.0% (133)	85.4% (70)	100.0% (15)
No committed relationship	8.0% (19)	5.0% (7)	14.6% (12)	0.0% (0)
Employment status				
Paid work (employment rate: ≥50%)	81.9% (194)	83.6% (117)	80.5% (66)	73.3% (11)
Paid work (employment rate: <50%)	8.4% (20)	10.7% (15)	6.1% (5)	0.0% (0)
No paid work	9.7% (23)	5.7% (8)	13.4% (11)	26.7% (4)
Nationality				
Swiss national	78.5% (186)	79.3% (111)	79.3% (65)	66.7% (10)
Foreign national	21.1% (50)	20.0% (28)	20.7% (17)	33.3% (5)
No answer	0.4% (1)	0.7% (1)	0.0% (0)	0.0% (0)

Although the gender ratio was balanced and the proportion of single parents corresponded roughly to that in the general population (14%; Federal Statistical Office, 2023b), it cannot be assumed that we have surveyed a representative sample of parents of minor children living in Switzerland. For example, at least compared to the general population (41%; Federal Statistical Office, 2023c), individuals with tertiary education were overrepresented in our sample (67%).

Regarding certain characteristics, we found significant differences between language regions. This was the case for the participants’ relationship status and their household income. Participants from the French-speaking regions were significantly less often in a committed relationship (85.4%; *n* = 70) than those from the German- (95.2%; *n* = 138) and Italian-speaking regions (100.0%; *n* = 10) (*p* = .021). Participants from the Italian-speaking region on the other hand were less able to get by on their household income (*p* = .001). These regional differences cannot be found in the general Swiss population. However, we have no data regarding these characteristics pertaining to parents of minor children.

### Measures

#### Sociodemographic characteristics

Socio-demographic questions included the respondents’ sex (female, male, divers), age, nationality (Swiss or foreign nationality), current employment status (paid work and employment rate/no paid work), educational status (compulsory education, upper secondary education, tertiary level education), relationship status (committed relationship: yes/no), status as a single parent (yes/no), household income (“getting by comfortably on household income”, “getting by on household income”, “difficult to get by on household income”, “very difficult to get by on household income”), and total number of children as well as the children’s age.

#### Parental burnout risk

Parental burnout risk was measured using the 3-L-BPBS. Respondents were presented with the items in their chosen language and asked to rate all five items on a 3-point scale as proposed by Aunola et al. (2021) (“daily”, “once or twice a week”, “more seldom/never”). The total score was computed by summing scores on all items. Therefore, the total score could range from 0 to 10. Aunola et al. (2021) defined two alternative cut-off values for the BPBs—one less (cut-off value: 2) and one more conservative (cut-off value: 3), the latter providing a higher level of specificity (Aunola et al., 2021). However, since it is not yet clear if these cut-off values can be applied to identifying parents at risk for parental burnout in Switzerland, we abstained from using them. Instead, we divided the sample into three groups: parents who have scored 0‒3 points (i.e., 30% of the maximum possible score; considered “probably no to low risk”), those with scores of 4‒7 (classified as “probably moderate risk”), and those with scores of 8‒10 points (labelled as “probably high risk”). In addition, we dichotomized categories as “probably no to low risk” (0‒3 points) and “probably moderate to high risk” (4‒10 points). In comparison to Aunola et al.’s (2021) cut-off values, our approach was more conservative in grouping parents.

#### Life satisfaction and satisfaction with family life

For assessing participants’ life satisfaction and satisfaction with family life, we used two slightly adapted items developed by Hipp et al. (2020). Participants were asked to indicate on a 7-point scale ranging from “very dissatisfied” (0) to “very satisfied” (6) how satisfied they were “with their life in general” or “with their family life” at the moment. The original items were in German, they were translated into French and Italian in the same manner as the 3-L-BPBS (see above).

#### Depression and sleep disorders

For assessing depressive symptoms, we used the Patient Health Questionnaire (PHQ-9), which is available in German, French, Italian, and various other languages (Bragantini, 2023; Löwe et al., 2002; Pfizer Inc., no date; Spitzer et al., no date). The PHQ-9 has good psychometric properties (Kroenke et al., 2001) and is widely used. Participants are asked to rate nine items on a 4-point Likert scale ranging from “not at all” (0) to “nearly every day” (3). Item scores are summed up, with higher scores indicating more depressive symptoms (Löwe et al., 2002). The scale’s internal consistency was good (α = .893). The PHQ-9 item “Trouble falling or staying asleep, or sleeping too much” (Löwe et al., 2002) was used as an indicator for sleep disorders. Here, the score could vary between 0 (“not at all”) and 3 (“nearly every day”).

#### Perceived burden of caring for children or other family members

Using the corresponding item from the Patient Health Questionnaire (PHQ-D; Löwe et al., 2002), respondents were asked to indicate how stressed they felt caring for their children or other family members on a 3-point scale ranging from “not stressed at all” (0) to “highly stressed” (2). The item’s German, French and Italian version were taken from the Swiss Health Survey (Federal Statistical Office, 2023d).

#### Social support

Social support was measured using the Oslo Social Support Scale (OSSS-3) (Bøen et al., 2012; Kocalevent et al., 2018). Respondents were asked to rate all three items of the OSSS-3. The sum score ranges from 3 to 14, with high values representing higher levels and low values representing lower levels of social support. Again, different language versions were taken from the Swiss Health Survey (Federal Statistical Office, 2023d). However, the scale’s internal consistency was questionable (α = 0.605).

### Analysis strategy

First, scale analyses were carried out for the 3-L-BPBS across language versions and for the German (3-L-BPBS-DE) and French language versions (3-L-BPBS-FR) separately. Since only 15 participants completed the Italian version (3-L-BPBS-IT), we abstained from carrying out a scale analysis for this version. Second, confirmatory factor analysis (CFA) was employed to assess the assumed unidimensionality of the 3-L-BPBS. To account for the categorical nature of the data, we utilized mean- and variance adjusted weighted least squares estimation (WLSMV) (for technical details, refer to Muthén et al., 1997). Research indicates that WLSMV performs well with small samples (*N* = 200) (e.g. Li, 2016). However, due to the tendency of chi-square to over-reject correct models in small samples (Li, 2016), alternative fit indices (AFI) were taken into account as well to investigate model fit, namely root mean square error of approximation (RMSEA), comparative fit index (CFI), Tucker-Lewis index (TLI), and the standardized root mean square residual (SRMR). Interpretation followed Hu and Bentler’s (1999) combinational rules (RMSEA < 0.06, SRMR < 0.07, CFI and TLI ≥ 0.95).

For the German and French language versions, we assessed measurement invariance using multi-group CFA, applying WLSMV estimation and multiple indicators multiple causal models (MIMIC) (Muthén and Muthén, 2017). We evaluated model fit for the configural (invariance of model form), weak (equivalence of factor loadings), and strong factorial invariance models (equivalence of factor loadings and item thresholds); the last two models were tested “by comparing the fit of two nested models that are identical except for a target set of restrictions in one” (Putnick and Bornstein, 2016: 8). We omitted the evaluation of strict factorial invariance (equivalence of items’ residuals), since it is not required for testing group mean differences (Putnick and Bornstein, 2016). To establish configural invariance, we used the following fit indices: CFI ≥ 0.95, RMSEA and SRMR < 0.08. Given the lack of consensus on optimal cut-off values for AFI when having ordinal data and small sample size (Putnick and Bornstein, 2016), we applied the commonly used criterion of a change in CFI of −0.01, combined with changes in RMSEA of 0.015 and SRMR of 0.030 or 0.015 (strong invariance), to evaluate the other forms of measurement invariance (Cheung and Rensvold, 2002; Putnick and Bornstein, 2016). Additionally, we looked at the significance of the change in chi-square. Although AFIs may over-reject correct models when evaluating absolute model fit in small samples, changes in AFIs appear to be less sensitive to sample size (*N* < 100) (Cheung and Rensvold, 2002; Putnick and Bornstein, 2016).

Third, Cronbach’s Alpha was used to examine the scale’s reliability. Again, analyses were carried out for the two language versions separately as well as for the 3-L-BPBS across languages. Lastly, we used Spearman correlations to test associations between the 3-L-BPBS total score and the following characteristics, which—according to previous studies (e.g. Aunola et al., 2021; Mikolajczak et al., 2018a)—should be related to parental burnout (construct validity): life satisfaction, satisfaction with family life, perceived burden of caring for children or other family members, depression, sleep disorders, and social support. In the case of significant correlations, we compared parents probably at moderate to high risk for parental burnout and parents probably not at risk or at low risk, using Mann-Whitney test to examine possible mean rank differences. Regarding the above-mentioned characteristics, the same test was used to analyse differences between participants using different language versions or living in different language regions. Effect sizes were interpreted according to Cohen (1992). Non-parametric tests were used due to non-normality of the data and to the ordinal level of most of the variables (e.g. sleep disorders; Agresti and Finlay, 2009). All analyses were carried out using IBM SPSS Statistics Version 29 and MPlus 8.9 (Muthén and Muthén, 2017).

## Results

In the following section, we will first present the results of scale and confirmatory factor analyses (CFA) for the 3-L-BPBS across language versions and for the German (3-L-BPBS-DE) and French (3-L-BPBS-FR) version separately. Then we will report estimated initial prevalence rates for parental burnout risk for Switzerland and for different language regions.

### Scale and factor analysis of the 3-L-BPBS (across language versions)

CFA results indicate an acceptable model fit (see Table 2). However, removing item 4 from the model (“I’m no longer able to show my child(ren) how much I love them”), which has the lowest difficulty index (0.08; see below) and the lowest factor loading (see Table 3), yielded better model fit. For both 3-L-BPBS versions CFI and TLI values were high (>0.95) and SRMR values low (<0.07). However, RMSEA values were above 0.06 and chi-squares were significant (see Table 2). Nevertheless, studies suggest that RMSEA and chi-square can be overly sensitive to small sample sizes (Lei and Shiverdecker, 2020; Li, 2016). Therefore, the overall model fit of the original and the 4-item model can be considered acceptable. For both 3-L-BPBS versions, standardized factor loadings in Table 3 indicate good validity of the construct “parental burnout risk”. However, it is important to note that the items included in the shortened 3-L-BPBS represent only two of the three characteristics associated with parental burnout: emotional exhaustion and feeling fed up with parenting. According to Roskam et al. (2018a), item 4 was the sole item representing parents’ emotional distancing from their children.

**Table 2.** Goodness-of-fit indicators of models for 3-L-BPBS across languages (5-item and 4-item 3-L-BPBS) (N = 237).

	X^2^ (df)	RMSEA (90%CI)	SRMR	CFI	TLI
3-L-BPBS (across languages) (5 items)	25.088 (5)***	0.130 (0.082-0.183)	0.044	0.988	0.976
3-L-BPBS (across languages) (4 items)	6.261 (2)*	0.098 (0.014-0.189)	0.018	0.997	0.992

***p < .001; **p < .01; ***p < .05.

**Table 3.** Unstandardized loadings (standard errors [S.E.]) and standardized loadings (S.E.) for confirmatory models of the 5-item and 4-item 3-L-BPBS (N = 237).

	5-item 3-L-BPBS		4-item 3-L-BPBS	
	Unstandardized loadings (S.E.)	Standardized loadings (S.E.)	Unstandardized loadings (S.E.)	Standardized loadings (S.E.)
Item 1: “I’m so tired out by my role as a parent that sleeping doesn’t seem like enough.”	1.000 (0.000)	0.883 (0.031)	1.000 (0.000)	0.893 (0.031)
Item 2: “I have the sense that I’m really worn out as a parent.”	1.053 (0.053)	0.930 (0.026)	1.078 (0.053)	0.963 (0.023)
Item 3: “I have the impression that I’m looking after my child(ren) on autopilot (I do what I’m supposed to do for my child(ren), but nothing more).”	0.938 (0.057)	0.828 (0.041)	0.932 (0.054)	0.833 (0.041)
Item 4: “I’m no longer able to show my child(ren) how much I love them.”	0.867 (0.066)	0.766 (0.053)	--	--
Item 5: “I feel like I can’t take any more as a parent.”	1.025 (0.049)	0.905 (0.030)	0.976 (0.054)	0.872 (0.038)

The response format was fully utilised and the distribution left-sloping for all five items. Items’ difficulty indices were low (0.08–0.17) which was to be expected, as the screening tool is intended to capture a rather rare construct. Variation of indices might be due to the fact that—according to Roskam and Mikolajczak (2021)—parental burnout first manifests itself in symptoms of exhaustion (items’ difficulty indices: 0.16–0.17) before parents report emotional distancing and feelings of being fed up with parenting (items’ difficulty indices: 0.08–0.11).

Cronbach’s Alpha of the original scale was 0.845, which is good regarding the small number of items. In addition, it is comparable to reliabilities reported by Aunola et al. (2021) (α = 0.81–0.86). Mean-Inter-Item-Correlation (MIC) was 0.52 and thus rather high. Item correlations varied between 0.32 and 0.72, and variance of the correlations was low (0.01). The part-whole corrected discriminatory power varied between 0.50 and 0.73. Thus, no item had low discriminatory power. Reliability analysis yielded better results for the shortened scale. Cronbach’s Alpha (α = 0.85) and MIC (0.59) of the 4-item scale were slightly higher. Item correlations were also higher and varied between 0.51 and 0.72, variance of correlations was low (0.00). Part-whole corrected discriminatory power varied between 0.64 and 0.76.

With regard to construct validity, correlations were calculated between original 3-L-BPBS scores and the above-mentioned characteristics which—according to previous studies (e.g. Aunola et al., 2021; Mikolajczak et al., 2018a)—should be related to parental burnout (see Table 4). As expected, parents probably at moderate to high risk for parental burnout were significantly less satisfied with their lives and their family life than parents probably not at risk or at low risk. Further, parents probably at moderate to high risk reported significantly more sleep disorders than parents at most at low risk, felt more stressed by caring for children or other family members, and showed more symptoms of depression. In addition, parents at moderate to high risk for parental burnout reported significantly less social support than parents probably at no to low risk. However, effect sizes were small to large (Cohen, 1992) (see Table 4).

**Table 4.** Correlations of parental burnout risk scores (original and 4-item scale) and selected characteristics of participants as well as summary of differences between parents probably at moderate to high risk and probably not at risk or low risk for parental burnout (Mann-Whitney U Test).

	3-L-BPBS (original)			3-L-BPBS (4-item scale)		
	r_S_	p (one-tailed)	*n*	r_S_	p (one-tailed)	*n*
Life satisfaction	−.266	<.001	235	−.254	<.001	235
Satisfaction with family life	−.325	<.001	237	−.313	<.001	237
Perceived burden of caring for children or other family members	.456	<.001	230	.467	<.001	230
Depression	.591	<.001	237	.586	<.001	237
Sleep disorders	.369	<.001	237	.376	<.001	237
Social support	−.287	<.001	224	−.299	<.001	224

	no/low risk (*n* = 191-201)	moderate/high risk (*n* = 33-36)			no/low risk (*n* = 180-189)	moderate/high risk (*n* = 44-48)	z value (p) (one-tailed)	r
	Mean rank (Mdn)	Mean rank (Mdn)	z value (p) (one-tailed)	r	Mean rank (Mdn)	Mean rank (Mdn)		
Life satisfaction	125.28 (5.00)	76.41 (4.00)	−4.138 (<.001)	.27	124.66 (5.00)	90.62 (5.00)	−3.213 (<.001)	.21
Satisfaction with family life	126.77 (5.00)	75.63 (4.00)	−4.355 (<.001)	.28	127.88 (5.00)	84.02 (4.00)	−4.182 (<.001)	.27
Perceived burden of caring for children or other family members	106.18 (1.00)	167.40 (2.00)	−5.368 (<.001)	.35	102.82 (1.00)	164.86 (2.00)	−6.108 (<.001)	.40
Depression	104.68 (4.00)	198.97 (10.13)	−7.625 (<.001)	.50	100.89 (4.00)	190.32 (10.06)	−8.098 (<.001)	.53
Sleep disorders	111.90 (1.00)	158.64 (1.00)	−4.064 (<.001)	.26	109.06 (1.00)	158.14 (1.00)	−4.778 (<.001)	.31
Social support	119.67 (10.00)	70.98 (9.00)	−4.036 (<.001)	.27	121.00 (10.00)	77.73 (9.00)	−4.021 (<.001)	.27

Interestingly, the same correlations were found for the 4-item 3-L-BPBS, indicating its construct validity even though the item representing the aspect of emotional distancing from one’s children is missing from the shortened scale (see Table 4). To examine differences between parents probably at risk and those probably not at risk, 4-item BPBS scores were dichotomized as “probably not at risk or low risk” (0-3 points; i.e., one third of the maximum possible scores) and “probably at moderate to high risk” (4-8 points).

**Figure 1.**
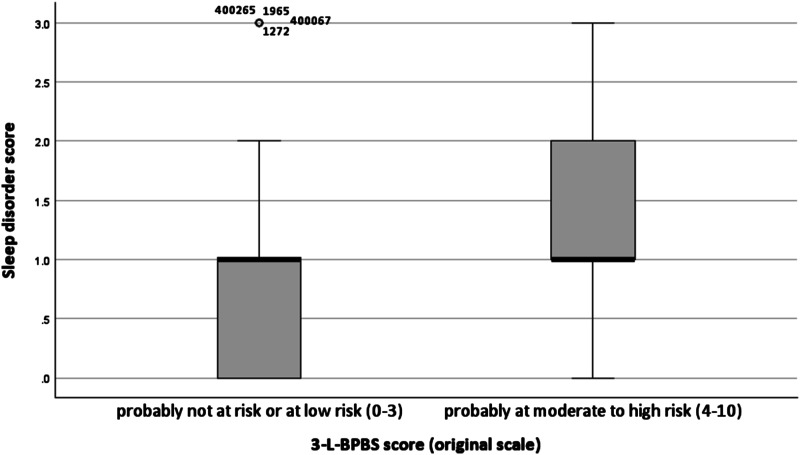
Box plots of sleep disorder score for parents probably at moderate to high risk for parental burnout and parents at most at low risk.

Regarding the results of group comparisons reported in Table 4, it should be noted that rank-sum tests, such as the Mann-Whitney test, utilize rankings of the observations and compare sample mean ranks (Agresti and Finlay, 2009). Therefore, significant differences between the analysed groups can be found, even when median values match. Figure 1 displays the results of the Mann-Whitney test, examining differences in sleep disorders between parents probably at moderate to high risk for parental burnout and parents at most at low risk. Whiskers show minimum and maximum values, boxes represent quartiles, and thicker horizontal lines indicate medians. Variation in dispersions of the data illustrates differences found between the two groups.

### Scale and multi-group confirmatory factor analyses of the original and the shortened 3-L-BPBS-DE and 3-L-BPBS-FR

Due to the better model fit of the 4-item 3-L-BPBS, we conducted multi-group CFA for the original and the shortened scale. Table 5 presents the results of the measurement invariance tests. Regarding the original 5-item 3-L-BPBS, configural invariance was achieved (see Table 5). However, weak factorial invariance was not achieved (*Χ*^*2*^_*diff*_(4) = 12.268, *p* = .016). Modification indices (M.I.) indicated that item 1 had the largest expected drop in the chi-square fit statistic (M.I. = 17.401) if factor loadings for this item were freed. Thus, only partial weak factorial invariance was achieved (*Χ*^*2*^_*diff*_(3) = 2.305, *p* = .512). In other words, both language groups exhibited the same pattern of factor loadings on the latent construct “risk for parental burnout,” but not all items contributed to the latent construct to a similar degree across language groups. Further, only partial strong invariance was achieved after thresholds for the first three items were freely estimated in both language groups and factor loadings for these items were freed (see Table 5). However, this means that the majority of items were noninvariant on the factor “parental burnout risk.” In addition, chi square difference test indicates that the restricted model had a poorer model fit (*Χ*^*2*^_*diff*_(2) = 27.500, *p* = .000). Thus, even though “no suggestion for an ‘acceptable’ proportion of invariant items” (Putnick and Bornstein, 2016: 9) has been proposed yet, we cannot readily assume partial strong invariance for the original 3-L-BPBS.

**Table 5.** Tests of measurement invariance of the original 5-item and the 4-item 3-L-BPBS.

	5-item 3-L-BPBS								
Model	X^2^(df)	CFI	RMSEA (90% CI)	SRMR	Model comp	ΔCFI	ΔRMSEA	ΔSRMR	Decision
M1 Configural invariance	35.300 (10)^***^	0.984	0.151 (0.099-0.207)	0.063	--	--	--	--	--
M2 Weak factorial invariance	41.859 (14)^***^	0.983	0.134 (0.088-0.182)	0.083	M1	0.001	0.017	0.020	rejected
M2.A Partial weak factorial invariance	30.291 (13)^**^	0.989	0.109 (0.059-0.161)	0.062	M1	-0.005	0.042	0.001	accepted
M3 Strong invariance	85.574 (23)^***^	0.962	0.157 (0.122-0.193)	0.098	M2.A	0.027	0.048	0.036	rejected
M3.A Partial strong invariance	48.567 (15)^***^	0.979	0.142 (0.099-0.188)	0.073	M2.A	0.010	0.033	0.011	accepted

	4-item 3-L-BPBS								
Model	X^2^(df)	CFI	RMSEA (90% CI)	SRMR	Model comp	ΔCFI	ΔRMSEA	ΔSRMR	Decision
M1 Configural invariance	7.556 (4)	0.998	0.089 (0.000-0.187)	0.023	--	--	--	--	--
M2 Weak factorial invariance	11.946 (7)	0.997	0.080 (0.000-0.155)	0.038	M1	−0.001	0.009	0.015	accepted
M3 Strong invariance	46.459 (15)^***^	0.978	0.137 (0.094-0.183)	0.069	M2	−0.019	0.057	0.031	rejected
M3.A Partial strong invariance	24.468 (8)^**^	0.988	0.136 (0.077-0.199)	0.042	M2	−0.009	0.056	0.004	accepted

^***^p < .001; ^**^ *p* < .01; * *p* < .05.

Results for the 4-item 3-L-BPBS are different. Here, configural and weak factorial invariance (*Χ*^*2*^_*diff*_(3) = 5.232, *p* = .156) were achieved. However, strong factorial invariance was not achieved (*Χ*^*2*^_*diff*_(8) = 32.495, *p* = .000). Modification indices indicated that item 3 had the largest expected drop in the chi-square fit statistic (M.I. = 30.189) if the threshold for this item is freely estimated in both language groups. Additionally, the equality constraint for item 2 was removed and factor loadings for items 2 and 3 were freed. Thus, only partial strong factorial invariance was achieved (*Χ*^*2*^_*diff*_(1) = 2.094, *p* = .148) (see Table 5).

Reliabilities of the two original 5-item language versions were high (α_German_ = 0.84; α_French_ = 0.85). The MIC was 0.50 for the German and 0.53 for the French version, which, again, is rather high. Item correlations varied between 0.15 and 0.64 for the German and between 0.30 and 0.78 for the French version. The lowest correlations were observed between item 4 and item 1 (3-L-BPBS-DE: 0.15) and between item 4 and item 3 (3-L-BPBS-FR: 0.30). The remaining values were significantly above 0.30, which is considered acceptable. Variance of the correlations was low (0.03 or 0.02). While it was expected that removing item 4 from the 3-L-BPBS-FR would increase Cronbach’s Alpha (α = 0.87), this was not observed in the scale’s German version (α = 0.83). Instead, removing item 1 led to a slight increase in Alpha (α = 0.84). No item had low discriminant power. The part-whole corrected discriminant power varied between 0.49 and 0.74 (German version) or 0.43 and 0.77 (French version). The shortened language versions demonstrated slightly better results: Reliabilities (α_German_ = 0.83; α_French_ = 0.87) and MIC were high (MIC_German_: 0.54; MIC_French_: 0.63). Item correlations varied between 0.36 and 0.64 for the German and between 0.55 and 0.78 for the French version. Variance of the correlations was low (in each case 0.01). The part-whole corrected discriminant power varied between 0.55 and 0.78 (German version) or 0.64 and 0.79 (French version).

Regarding construct validity, the results for the French and German 5-item and 4-item versions were largely comparable with those of the 3-L-BPBS across language versions (see Table 6). However, while in both German versions social support and satisfaction with family life were significantly but weakly correlated with parental burnout risk, these negative correlations were stronger in the French versions (see Table 6).

**Table 6.** Correlations of parental burnout risk scores (5-item 3-L-BPBS-DE and 3-L-BPBS-FR; 4-item 3-L-BPBS-DE and 3-L-BPBS-FR) and selected characteristics of participants as well as summary of differences between parents probably at moderate to high risk and probably not at risk or low risk for parental burnout (Mann-Whitney U Test).

	5-item 3-L-BPBS-DE			5-item 3-L-BPBS-FR		
	*r_S_*	*p (one-tailed)*	*n*	*r_S_*	*p (one-tailed)*	*n*
Life satisfaction	−.319	.000	139	−.308	.003	81
Satisfaction with family life	−.267	.001	140	−.472	<.001	82
Perceived burden of caring for children or other family members	.384	.000	134	.548	<.001	81
Depression	.612	.000	140	.596	<.001	82
Sleep disorders	.376	.000	140	.478	<.001	82
Social support	−.219	.005	135	−.382	<.001	77

	no/low risk (n = 121-126)	moderate/high risk (n = 13-14)			no/low risk (n = 58-61)	moderate/high risk (n = 19-21)		
	*Mean rank (Mdn)*	*Mean rank (Mdn)*	*z value (p) (one-tailed)*	*r*	*Mean rank (Mdn)*	*Mean rank (Mdn)*	*z value (p) (one-tailed)*	*r*
Life satisfaction	72.24 (5.00)	48.27 (4.00)	−2.185 (.015)	.19	45.60 (5.00)	27.86 (4.00)	−3.095 (<.001)	.34
Satisfaction with family life	73.05 (5.00)	47.54 (4.50)	−2.390 (.009)	.20	46.56 (6.00)	26.81 (4.00)	−3.522 (<.001)	.39
Perceived burden of caring for children or other family members	64.20 (1.00)	98.19 (2.00)	−3.233 (<.001)	.28	35.60 (1.00)	56.43 (2.00)	−3.729 (<.001)	.41
Depression	64.47 (4.00)	124.75 (11.00)	−5.300 (<.001)	.45	34.41 (5.00)	62.10 (10.13)	−4.606 (<.001)	.51
Sleep disorders	68.36 (1.00)	89.79 (1.00)	−2.026 (.024)	.17	36.48 (1.00)	56.07 (1.00)	−3.482 (<.001)	.38
Social support	70.05 (11.00)	50.32 (9.50)	−1.812 (.035)	.16	44.02 (10.00)	23.68 (8.00)	−3.477 (<.001)	.40

	4-item 3-L-BPBS-DE			4-item 3-L-BPBS-FR		
	*r*_*S*_	*p (one-tailed)*	*n*	*r*_*S*_	*p (one-tailed)*	*n*
Life satisfaction	−.313	<.001	139	−.280	.006	81
Satisfaction with family life	−.263	<.001	140	−.437	<.001	82
Perceived burden of caring for children or other family members	.378	<.001	134	.569	<.001	81
Depression	.606	<.001	140	.593	<.001	82
Sleep disorders	.376	<.001	140	.486	<.001	82
Social support	−.219	.005	135	−.402	<.001	77

	no/low risk (n = 117-122)	moderate/high risk (n = 17-18)			no/low risk (n = 50-53)	moderate/high risk (n = 27-29)		
	*Mean rank (Mdn)*	*Mean rank (Mdn)*	*z value (p) (one-tailed)*	*r*	*Mean rank (Mdn)*	*Mean rank (Mdn)*	*z value (p) (one-tailed)*	*r*
Life satisfaction	72.48 (5.00)	52.24 (5.00)	−2.076 (.019)	.18	44.74 (5.00)	33.93 (5.00)	−2.046 (.020)	.23
Satisfaction with family life	73.27 (5.00)	51.69 (5.00)	−2.255 (.012)	.19	48.05 (6.00)	29.53 (4.00)	−3.617 (<.001)	.40
Perceived burden of caring for children or other family members	62.95 (1.00)	98.79 (2.00)	−3.834 (<.001)	.33	33.27 (1.00)	54.86 (2.00)	−4.229 (<.001)	.47
Depression	63.53 (3.50)	117.75 (10.00)	−5.319 (<.001)	.45	31.35 (4.00)	60.05 (10.13)	−5.230 (<.001)	.58
Sleep disorders	67.72 (1.00)	89.36 (1.00)	−2.283 (.013)	.19	33.97 (1.00)	55.26 (1.00)	−4.145 (<.001)	.46
Social support	70.57 (11.00)	50.18 (9.00)	−2.039 (.020)	.18	44.83 (10.00)	28.20 (8.00)	−3.147 (<.001)	.36

In this regard, it is also interesting that we found significant differences between language regions regarding participants’ perceived burden of caring for children or other family members and depressive symptoms. Parents from the French-speaking regions tended to feel slightly more burdened by caring responsibilities (*Mdn =* 1.00) and showed more signs of depression (*Mdn* = 5.81) than parents from the German-speaking part of Switzerland (*Mdn* = 1.00, *z =* −1.910, *p* = .056, *r* = 0.13; *Mdn* = 4.00, *z* = −2.015, *p* = .044, *r* = 0.13). However, effect sizes were rather small (Cohen, 1992). No significant differences regarding life satisfaction (*z =* −0.208, *p* = .835), satisfaction with family life (*z* = −0.713, *p =* .476), social support (*z* = −1.247, *p* = 212), or sleep disorders (*z* = −0.684, *p* = .494) could be found.

### Initial estimation of prevalence rates of parental burnout risk in Switzerland

Respondents scored between 0 and 10 on the original 3-L-BPBS (*M* = 1.36, *Mdn* = 0.00, *SD* = 2.19), and between 0 and 8 on the shortened version (*M* = 1.20, *Mdn* = 0.00, *SD* = 1.93). Depending on the scale used, analyses yielded an initial prevalence rate of 15.2% parents probably at moderate to high risk for parental burnout (3-L-BPBS score ≥ 4; *n* = 36) or 20.3% (4-item 3-L-BPBS score ≥ 3; *n* = 48) in our sample of Swiss parents of minor children (*N* = 237).

**Figure 2.**
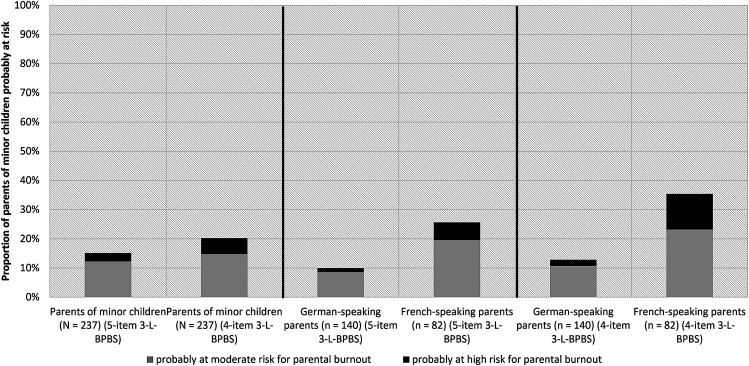
Percentage of Swiss parents of minor children probably at moderate or high risk for parental burnout in the sample, differentiated by scale version and by language.

Parental burnout risk frequencies differed significantly between the scale’s German- and French-speaking version. Whereas 10.0% (5-item 3-L-BPBS score ≥ 4; *n* = 14) or 12.9% (4-item 3-L-BPBS score ≥ 3; *n* = 18) of German-speaking parents were probably at moderate to high risk for parental burnout, this held true for 25.6% (5-item 3-L-BPBS score ≥ 4; *n* = 21) or 35.4% (4-item 3-L-BPBS score ≥ 3; *n* = 29) of French-speaking parents in our sample (see Figure 2). However, due to noninvariance of about half of the items, differences between both language groups must be interpreted with caution. In contrast to the German- and French-speaking respondents, only one of the surveyed parents from the Italian-speaking canton of Ticino reached a risk score of more than 4 on the original 5-item or more than 3 on the 4-item scale. However, due to the small sample size and because we could not test measurement invariance for the Italian version these results cannot be reliably interpreted.

In line with the findings for the German and French language versions, respondents’ parental burnout risk scores also differed significantly or tended to differ significantly between the two language regions (5-item 3-L-BPBS: *Χ*^*2*^(1) = 4.271, *p* = .054; 4-item 3-L-BPBS: *Χ*^*2*^(1) = 9.618, *p* = .002). Mann-Whitney test confirmed that parents from the German-speaking (5-item 3-L-BPBS: *Mdn* = 0.00, *Min* = 0, *Max* = 8, *n* = 142; 4-item 3-L-BPBS: *Mdn* = 0.00, *Min* = 0, *Max* = 8, *n* = 142) and the French-speaking part of Switzerland (5-item 3-L-BPBS: *Mdn* = 1.00, *Min* = 0, *Max* = 10, *n* = 80; 4-item 3-L-BPBS: *Mdn* = 1.00, *Min* = 0, *Max* = 8, *n* = 80) differed significantly regarding their parental burnout risk scores (5-item 3-L-BPBS: *z* = −3.636, *p* < .001; 4-item 3-L-BPBS: *z* = −3.438, *p* < .001).

## Discussion

Results indicate that a significant proportion of Swiss parents might be at least at risk for parental burnout. Depending on the scale used, 15–20% of parents of minor children were probably at moderate to high risk in our sample. This held especially true for parents in the French-speaking regions of Switzerland. Thus, initial prevalence rates for parental burnout risk found in the Swiss sample were higher than prevalence rates for parental burnout found in other countries (Roskam et al., 2021). This is due to three reasons: For one, we studied risk for parental burnout whereas Roskam et al. (2021) were interested in the prevalence of parental burnout. Secondly, we collected our data in a time when the COVID-19 pandemic and pandemic-related public health measures had accompanied the Swiss population for more than two years. This might have led to an increase in parents at risk for parental burnout. Accordingly, Van Bakel et al. (2022) found increased prevalence rates when comparing pre- and peri-pandemic parental burnout rates in different countries. Lastly, we focused on parents of minor children, whereas Roskam et al. (2021) included parents of children living at home regardless of their age.

Identifying parents at risk for parental burnout seems especially important when considering the dire consequences parental burnout can have on parents, children, and family systems. Regarding the children, early detection of parents at risk might prevent them being abused and/or neglected.

To measure parental burnout risk, we translated and adapted the Brief Parental Burnout Scale proposed by Aunola et al. (2021) to the Swiss context, developing the Trilingual Swiss Brief Parental Burnout Scale (3-L-BPBS). Overall, the psychometric properties of the 3-L-BPBS were satisfactory. Our results are consistent with those regarding the original BPBs (Aunola et al., 2021) and with previous findings on parental burnout (e.g. Aunola et al., 2020; Hamvai et al., 2022; Mikolajczak et al., 2019; Roskam and Mikolajczak, 2021). However, when testing the 3-L-BPBS’s factor structure, a 4-item scale yielded slightly better model fit than the original 5-item 3-L-BPBS. Although the psychometric properties of the 4-item scale were also satisfactory and consistent with previous findings, the shortened scale only comprises items that—according to Roskam et al. (2018a)—represent two of the three aspects associated with parental burnout: emotional exhaustion regarding the parenting role and the feeling of being fed up with parenting. However, having the impression of “looking after one’s children on autopilot” might not only imply emotional exhaustion but also an emotional distancing from the children. Results regarding the scale’s construct validity indicate that either all three aspects are represented despite the missing item or that Swiss parents might have a different understanding of parental burnout. Further research is required on this topic, particularly qualitative studies that involve participants from various language regions to explore their understanding of the items.

Furthermore, concerning the differences in parental burnout scores between German- and French-speaking respondents, the lack of strong invariance must be considered. In other words, we cannot readily assume that parental burnout risk measured with the original 5-item or the 4-item 3-L-BPBS has the same meaning for the surveyed German- and French-speaking parents, and differences between the two groups must be interpreted with caution. Both the deviation in factor structure and the violation of strong invariance, might simply be due to translation, the small sample size, or item 4 might have been a particularly sensitive item for respondents, making it more susceptible to socially desirable responses. Yet, these differences might also reflect cultural differences between Switzerland and the two countries in which the original BPBs was developed and validated, namely Belgium and Finland (Aunola et al., 2021). Aunola et al. (2021) themselves discuss possible cultural and societal effects on parental burnout and its measurement. However, more internationally comparative validation studies on the BPBS are needed here.

Yet, not only more internationally comparative studies on parental burnout risk and its measurement are needed. Our results suggest that we also need regionally comparative ones. This seems important because our results not only indicate significant differences in initial parental burnout risk prevalence rates between language versions but between parents living in different language regions of Switzerland as well. On the one hand, this was to be expected due to the very strong correlation between language version and language region. On the other hand, it seems plausible that structural and cultural differences between Swiss language regions affect parental burnout risk prevalence. For example, parents in French-speaking Switzerland are less likely to follow traditional gender role models regarding workload of mothers of young children or, as mentioned above, more likely to use extrafamilial childcare than parents in German-speaking regions (Federal Statistical Office, 2021). In addition, educational behaviour differs between language regions (Schöbi et al., 2020). To some extent these regional differences are due to the fact, that, culturally, French-speaking Switzerland in many aspects is oriented towards France (e.g. Bradley, 2020) whereas German-speaking regions are more oriented towards Germany. Thus, the fact that parental burnout prevalence rates reported for France (5.3–6.2%) equal those for the French-speaking canton of Vaud (3.2–7.1%), and both were higher than parental burnout prevalence for Germany (1.5–1.8%) (Roskam et al., 2021), can be seen as an additional indicator for the impact of structural and cultural characteristics of language regions on parental burnout.

Furthermore, differences between the two language regions might have been reinforced by the COVID-19 pandemic. French-speaking Switzerland was more affected by the virus itself and containment measures therefore much stricter than in German-speaking cantons (Moser et al., 2021). Against this backdrop, we assume that the reported differences in initial prevalence rates for parental burnout risk between the two language regions are due to structural and cultural differences between the regions and not to modifications in item meaning due to translation or differences in understanding of parental burnout risk. This is also supported by the study’s finding that parents from French-speaking cantons felt more burdened by care responsibilities and showed more depressive symptoms than respondents from German-speaking cantons. However, additional studies are needed to further examine differences between language regions.

### Limitations

Some limitations have to be taken into account when interpreting the study’s results. Overall, it should be noted that although the subsample of parents comes from a structurally representative sample of the Swiss population, this does not mean that it is representative of all parents in Switzerland. Even though certain characteristics in the sample approximate the general population proportions (e.g. participants’ gender), this does not apply for all characteristics (e.g. educational status). Further, we have a fundamental problem in assessing the representativeness of our subsample of parents of minor children: we lack data on this particular population group in Switzerland to compare our sample to. A further limitation lies in the use of a self-report measure which are subject to social desirability bias. However, the effect of social desirability should be minimized by the anonymous nature of the present study.

Regarding the identified differences in initial prevalence rates between language regions and the scale’s language versions, further research is needed to test our hypotheses about the effect of structural and cultural differences. Moreover, due to the small size of the Italian-speaking subsample, we were not able to validate the scale’s Italian version. Further, only partial strong invariance could be achieved, which might lead to serious misinterpretation of mean differences between language versions or regions (Putnick and Bornstein, 2016). In sum, a follow-up study is needed to further validate the 3-L-BPBS’s factor structure, psychometric properties, sensitivity, and specificity, and to determine appropriate cut-off values for Switzerland using a larger sample of parents. This is especially important because as Aunola et al. (2021) pointed out, it is possible, that sensitivity and specificity of the short scale may vary between countries due to cultural and societal effects and that the optimal cut-off values may differ across countries or cultures. Lastly, longitudinal studies are needed allowing to examine causal relationships between parents’ characteristics and parental burnout (risk). These would also allow for studying possible impacts of events such as the pandemic-related public health measures on parental burnout.

### Conclusion

The 3-L-BPBS seems to be a promising screening tool allowing early detection of parents at risk for parental burnout and thus the prevention of its adverse effects on parents’ and children’s health and family systems. Although further studies are needed to validate the instrument, an estimation of initial prevalence rates for parental burnout risk suggests that parental burnout might be a burning issue in Switzerland. Initial prevalence rates seem rather high, but not implausible, especially considering probable long-term effects of the COVID-19 pandemic on families. Further, the study suggests that not only differences between different countries have to be considered when researching parental burnout but also regional differences regarding family-related issues, such as compatibility of family and career or educational attitudes.

## References

[bibr1-20551029241308777] Agresti A Finlay B (2009) Statistical Methods for the Social Sciences. London: Pearson.

[bibr2-20551029241308777] Aunola K Sorkkila M Tolvanen A (2020) Validity of the Finnish version of the parental burnout assessment (PBA). Scandinavian Journal of Psychology 61(5): 714–722. 32542689 10.1111/sjop.12654

[bibr3-20551029241308777] Aunola K Sorkkila M Tolvanen A , et al. (2021) Development and validation of the brief parental burnout scale (BPBS). Psychological Assessment 33(11): 1125–1137. 34516161 10.1037/pas0001064

[bibr4-20551029241308777] Bøen H Dalgard OS Bjertness E (2012) The importance of social support in the associations between psychological distress and somatic health problems and socio-economic factors among older adults living at home: a cross sectional study. BioMed Central Geriatrics 12: 27. 22682023 10.1186/1471-2318-12-27 PMC3464708

[bibr5-20551029241308777] Bradley S (2020) How the Covid-19 crisis reveals cultural divide between Swiss language regions. In: swissinfo.ch. Available at: https://www.swissinfo.ch/eng/society/covid-19-impact_how-the-crisis-reveals-cultural-divide-between-swiss-language-regions/45647692 (accessed 12 December 2024).

[bibr6-20551029241308777] Bragantini F (2023) Yes-Men e Brownnosers in contesto lavorativo: preliminare valutazione delle proprietà psicometriche di strumenti di assessment ed esplorazione di sintomi ansiosi e depressivi. [Yes-Men and Brownnosers in work context: preliminary evaluation of psychometric properties of assessment instruments and exploration of anxiety and depressive symptoms]. Master Thesis, University of Padova, Italy. Available at: https://thesis.unipd.it/retrieve/7ebbd7fd-a792-4d3a-b6a7-60a1bc096d77/Bragantini_Fabio.pdf (accessed 12 December 2024).

[bibr8-20551029241308777] Cai H Xie X-M Zhang Q , et al. (2021) Prevalence of suicidality in major depressive disorder: a systematic review and meta-analysis of comparative studies. Frontiers in Psychiatry 12: 690130. 34603096 10.3389/fpsyt.2021.690130 PMC8481605

[bibr9-20551029241308777] Cheung GW Rensvold RB (2002) Evaluating goodness-of-fit indexes for testing measurement invariance. Structural Equation Modeling 9: 233–255.

[bibr10-20551029241308777] Cohen J (1992) A power primer. Psychological Bulletin 112(1): 155–159. 19565683 10.1037//0033-2909.112.1.155

[bibr11-20551029241308777] Diaz Hernandez L Giezendanner S Fischer R , et al. (2021) The effect of COVID-19 on mental well-being in Switzerland: a cross-sectional survey of the adult Swiss general population. BMC Family Practice 22(1): 181. 34507540 10.1186/s12875-021-01532-7 PMC8432273

[bibr12-20551029241308777] Edú-Valsania S Laguía A Moriano JA (2022) Burnout: a review of theory and measurement. International Journal of Environmental Research and Public Health 19: 1780. 35162802 10.3390/ijerph19031780 PMC8834764

[bibr13-20551029241308777] Favez N Max A Bader M , et al. (2023) When not teaming up puts parents at risk: coparenting and parental burnout in dual-parent heterosexual families in Switzerland. Family Process 62: 272–286. 35396850 10.1111/famp.12777

[bibr14-20551029241308777] Federal Statistical Office (2020) Familien- und schulergänzende Kinderbetreuung im Jahr 2018. [Extrafamilial and school supplementary childcare, 2018]. Report, no. 2019-1800, 25 May. Neuchâtel: Federal Statistical Office. Available at: https://www.bfs.admin.ch/bfs/de/home/statistiken/bevoelkerung/familien/familienergaenzende-kinderbetreuung.assetdetail.12867117.html (accessed 12 December 2024).

[bibr15-20551029241308777] Federal Statistical Office (2021) Familien in der Schweiz [Families in Switzerland]. Report, no 1010-2100, 11 May. Neuchâtel: Federal Statistical Office. Available at: https://www.bfs.admin.ch/bfs/en/home/statistics/population/effectif-change/regional-distribution.assetdetail.26565304.html (accessed 12 December 2023).

[bibr16-20551029241308777] Federal Statistical Office (2023a) Permanent resident population by age, canton, district and commune, 2010-2022. Available at: https://www.bfs.admin.ch/bfs/de/home/statistiken/bevoelkerung/stand-entwicklung/raeumliche-verteilung.assetdetail.26565304.html (accessed 12 December 2024).

[bibr17-20551029241308777] Federal Statistical Office (2023b) Einfamilienhaushalte mit mindestens einem Kind unter 18 Jahren, 2021 [Single-family households with at least one child under 18, 2021]. Available at: https://www.bfs.admin.ch/bfs/de/home/statistiken/bevoelkerung/familien/haushalte.assetdetail.24205241.html (accessed 12 December 2024).

[bibr18-20551029241308777] Federal Statistical Office (2023c) Höchste abgeschlossene Ausbildung nach verschiedenen soziodemografischen Merkmalen in der Schweiz, 2021 [Highest level of education completed differentiated by selected socio-demographic characteristics in Switzerland, 2021]. Available at: https://www.bfs.admin.ch/bfs/de/home/statistiken/bildung-wissenschaft/bildungsstand.assetdetail.23965914.html (accessed 12 December 2024).

[bibr19-20551029241308777] Federal Statistical Office (2023d) Schweizerische Gesundheitsbefragung [Swiss health survey]. Available at: https://www.bfs.admin.ch/bfs/de/home/statistiken/gesundheit/erhebungen/sgb.html#1243813114 (accessed 12 December 2024).

[bibr20-20551029241308777] Fuchs G Lanfranconi L Abbas M , et al. (2021) Nationales Barometer zur Gleichstellung 2021: Fokus Erwerbsarbeit und unbezahlte Care-Arbeit. [National Barometer on Gender Equality 2021: Focus on paid work and unpaid care work]. Report. Switzerland, November: Lucerne University of Applied Sciences and Arts – School of Social Work. Available at: https://www.equality.ch/pdf_d/Barometer_DE_komplett.pdf (accessed 12 December 2024).

[bibr21-20551029241308777] Furutani K Kawamoto T Alimardani M , et al. (2020) Exhausted parents in Japan: preliminary validation of the Japanese version of the Parental Burnout Assessment. New Directions for Child and Adolescent Development 2020(174): 33–49. 33029919 10.1002/cad.20371 PMC7821145

[bibr22-20551029241308777] Hamvai C Hidegkuti I Vargha A , et al. (2022) Parental burnout in Hungary: development and psychometric evaluation of the Hungarian parental burnout assessment (PBA-HUN). European Journal of Mental Health 17(1): 47–61.

[bibr23-20551029241308777] Hansotte L Nguyen N Roskam I , et al. (2021) Are all burned out parents neglectful and violent? A latent profile analysis. Journal of Child and Family Studies 30(1): 158–168.

[bibr24-20551029241308777] Hipp L Bünning M Munnes S (2020) Codebook Coronabefragung: Welle 1.1: 23.03.2020–05.04.2020. [Codebook Corona Survey: Wave 1.1: 23.03.2020–05.04.2020]. Report, Berlin Social Science Center, Germany, April. Available at: https://www.wzb.eu/system/files/docs/dsi/af/codebook_1.1_deu_0.pdf (accessed 12 December 2024).

[bibr25-20551029241308777] Hu L Bentler PM (1999) Cutoff criteria for fit indexes in covariance structure analysis: conventional criteria versus new alternatives. Structural Equation Modeling: A Multidisciplinary Journal 6(1): 1–55.

[bibr26-20551029241308777] Kocalevent R-D Berg L Beutel ME , et al. (2018) Social support in the general population: standardization of the Oslo social support scale (OSSS-3). BMC Psychology 6(1): 31. 30016997 10.1186/s40359-018-0249-9 PMC6050647

[bibr27-20551029241308777] Kroenke K Spitzer RL Williams JB (2001) The PHQ-9: validity of a brief depression severity measure. Journal of General Internal Medicine 16(9): 606–613. 11556941 10.1046/j.1525-1497.2001.016009606.x PMC1495268

[bibr28-20551029241308777] Krüger P Caviezel Schmitz S (2022) Family violence and COVID-19: the pandemic within the pandemic. Study Protocol, Unpublished manuscript.

[bibr29-20551029241308777] Lei P-W Shiverdecker LK (2020) Performance of estimators for confirmatory factor analysis of ordinal variables with missing data. Structural Equation Modeling: A Multidisciplinary Journal 27(4): 584–601.

[bibr30-20551029241308777] Li CH (2016) Confirmatory factor analysis with ordinal data: comparing robust maximum likelihood and diagonally weighted least squares. Behavior Research Methods 48: 936–949. 26174714 10.3758/s13428-015-0619-7

[bibr31-20551029241308777] Löwe B Spitzer RL Zipfel S , et al. (2002) PHQ-D Gesundheitsfragebogen für Patienten [German Version of the Patient Health Questionnaire PHQ-D]. Karlsruhe: Pfizer.

[bibr32-20551029241308777] Matias M Aguiar J César F , et al. (2020) The Brazilian–Portuguese version of the Parental Burnout Assessment: transcultural adaptation and initial validity evidence. New Directions for Child and Adolescent Development 2020(174): 67–83. 33084172 10.1002/cad.20374

[bibr33-20551029241308777] Mikolajczak M Brianda ME Avalosse H , et al. (2018a) Consequences of parental burnout: its specific effect on child neglect and violence. Child Abuse & Neglect 80: 134–145. 29604504 10.1016/j.chiabu.2018.03.025

[bibr34-20551029241308777] Mikolajczak M Raes M-E Avalosse H , et al. (2018b) Exhausted parents: sociodemographic, child-related, parent-related, parenting and family-functioning correlates of parental burnout. Journal of Child and Family Studies 27(2): 602–614.

[bibr35-20551029241308777] Mikolajczak M Gross JJ Roskam I (2019) Parental burnout: what is it, and why does it matter? Clinical Psychological Science 7(6): 1319–1329.

[bibr36-20551029241308777] Mikolajczak M Gross JJ Stinglhamber F , et al. (2020) Is parental burnout distinct from job burnout and depressive symptoms? Clinical Psychological Science 8(4): 673–689.

[bibr37-20551029241308777] Mikolajczak M Gross JJ Roskam I (2021) Beyond job burnout: parental burnout. Trends in Cognitive Sciences 25(5): 333–336. 33714668 10.1016/j.tics.2021.01.012

[bibr38-20551029241308777] Moser A von Wyl V Höglinger M (2021) Health and social behaviour through pandemic phases in Switzerland: regional time-trends of the COVID-19 Social Monitor panel study. PLoS One 16(8): e0256253. 34432842 10.1371/journal.pone.0256253 PMC8386858

[bibr39-20551029241308777] Muthén LK Muthén BO (2017) Mplus: Statistical Analysis with Latent Variables: User’s Guide (Version 8). Los Angeles, CA: Authors.

[bibr40-20551029241308777] Muthén BO du Toit SHC Spisic D (1997) Robust inference using weighted least squares and quadratic estimating equations in latent variable modeling with categorical and continuous outcomes. Available at: https://www.statmodel.com/download/Article_075.pdf.

[bibr41-20551029241308777] Oh DJ Shin YC Oh KS , et al. (2023) Examining the links between burnout and suicidal ideation in diverse occupations. Frontiers in Public Health 11: 1243920. 37744483 10.3389/fpubh.2023.1243920 PMC10513409

[bibr42-20551029241308777] Pfizer Inc . (no date). Patient health questionnaire screener. Available at:. https://www.phqscreeners.com/ (accessed 12 December 2024).

[bibr43-20551029241308777] Prandstetter K Murphy H Foran HM (2023) The role of intimate partner violence, couple dissatisfaction and parenting behaviors in understanding parental burnout. Journal of Child and Family Studies 32(1): 343–355. 35068912 10.1007/s10826-021-02218-5 PMC8760085

[bibr44-20551029241308777] Putnick DL Bornstein MH (2016) Measurement invariance conventions and reporting: the state of the art and future directions for psychological research. Developmental Review 41: 71–90. 27942093 10.1016/j.dr.2016.06.004 PMC5145197

[bibr45-20551029241308777] Refle J-E Voorpostel M Lebert F , et al. (2020) First results of the Swiss household panel – COVID-19 study. FORS Working Paper Series No 2020-1, (no date). Lausanne: FORS. Available at: https://forscenter.ch/working-papers/fwp-2020-00001/ (accessed 15 December 2023).

[bibr47-20551029241308777] Roskam I Mikolajczak M (2021) The slippery slope of parental exhaustion: a process model of parental burnout. Journal of Applied Developmental Psychology 77: 101354.

[bibr48-20551029241308777] Roskam I Raes M-E Mikolajczak M (2017) Exhausted parents: development and preliminary validation of the parental burnout inventory. Frontiers in Psychology 8: 163. 28232811 10.3389/fpsyg.2017.00163 PMC5298986

[bibr49-20551029241308777] Roskam I Brianda M-E Mikolajczak M (2018a) A step forward in the conceptualization and measurement of parental burnout: the Parental Burnout Assessment (PBA). Frontiers in Psychology 9: 758. 29928239 10.3389/fpsyg.2018.00758 PMC5998056

[bibr50-20551029241308777] Roskam I Raes M-E Mikolajczak M (2018b) Corrigendum: “exhausted parents: development and preliminary validation of the parental burnout inventory”. Frontiers in Psychology 9: 73. 29403419 10.3389/fpsyg.2018.00073 PMC5797643

[bibr51-20551029241308777] Roskam I Aguiar J Akgun E , et al. (2021) Parental burnout around the globe: a 42-country study. Affective Science 2(1): 58–79. 33758826 10.1007/s42761-020-00028-4 PMC7970748

[bibr52-20551029241308777] Roskam I Bayot M Mikolajczak M (2022) Parental burnout assessment (PBA). In: Medvedev ON Krägeloh CU Siegert RJ , et al. (eds) Handbook of Assessment in Mindfulness Research. Cham: Springer International Publishing, 1–22.

[bibr53-20551029241308777] Schöbi B Holmer P Rapicault A , et al. (2020) Bestrafungsverhalten von Eltern in der Schweiz. [Punitive behaviour of parents in Switzerland]. Report, University of Fribourg, Switzerland, Available at: https://www.kinderschutz.ch/angebote/herunterladen-bestellen/studie-bestrafungsverhalten-eltern-2020?gclid=EAIaIQobChMI1peXq7uXgwMV0aiDBx0sEQKZEAAYASAAEgIOYvD_BwE (accessed 12 December 2024).

[bibr54-20551029241308777] Sorkkila M Aunola K (2020) Risk factors for parental burnout among Finnish parents: the role of socially prescribed perfectionism. Journal of Child and Family Studies 29(3): 648–659.

[bibr55-20551029241308777] Spitzer RL Williams JBW Kroenke K (no date) Questionnaire sur la santé du patient – 9 (PHQ-9) [Patient Health Questionnaire PHQ-9]. Available at: https://www.prevention-depression.lu/wp-content/uploads/PHQ9_French_for_France.pdf (accessed 12 December 2024).

[bibr56-20551029241308777] Szczygieł D Sekulowicz M Kwiatkowski P , et al. (2020) Validation of the polish version of the parental burnout assessment (PBA). New Directions for Child and Adolescent Development 2020(174): 137–158. 33201567 10.1002/cad.20385

[bibr57-20551029241308777] van Bakel H Bastiaansen C Hall R , et al. (2022) Parental burnout across the globe during the COVID-19 pandemic. International Perspectives in Psychology 11(3): 141–152.

